# Toward a Unified Framework for Positive Psychology Interventions: Evidence-Based Processes of Change in Coaching, Prevention, and Training

**DOI:** 10.3389/fpsyg.2021.809362

**Published:** 2022-02-10

**Authors:** Joseph Ciarrochi, Steven C. Hayes, Lindsay G. Oades, Stefan G. Hofmann

**Affiliations:** ^1^Institute of Positive Psychology and Education, Australian Catholic University, Sydney, NSW, Australia; ^2^Department of Psychology, University of Nevada, Reno, NV, United States; ^3^Centre for Positive Psychology, University of Melbourne, Melbourne, VIC, Australia; ^4^Department of Psychological and Brain Sciences, Philipps University Marburg, Marburg, Germany; ^5^Boston University, Boston, MA, United States

**Keywords:** positive psychology, process-based coaching, training, therapy, extended evolutionary meta-model, mediation

## Abstract

Since 2000, research within positive psychology has exploded, as reflected in dozens of meta-analyses of different interventions and targeted processes, including strength spotting, positive affect, meaning in life, mindfulness, gratitude, hope, and passion. Frequently, researchers treat positive psychology processes of change as distinct from each other and unrelated to processes in clinical psychology. This paper presents a comprehensive framework for positive psychology processes that crosses theoretical orientation, links coherently to clinical psychology and its more dominantly “negative” processes, and supports practitioners in their efforts to personalize positive psychological interventions. We argue that a multi-dimensional and multi-level extended evolutionary approach can organize effective processes of change in psychosocial interventions, by focusing interventions on context-appropriate variation, selection, and retention of processes, arranged in terms of key biopsychosocial dimensions across psychological, biophysiological, and sociocultural levels of analysis. We review widely studied positive psychology constructs and programs and show how this evolutionary approach can readily accommodate them and provide a common language and framework for improving human and community flourishing. We conclude that Interventions should start with the person, not the protocol.

In 2000, Seligman and Csikszentmihalyi argued that psychology had become excessively focused on negative traits, pathology, and repairing psychological damage, and had neglected the study of individual and community flourishing. Since that time, the field of Positive Psychology has thrived, with thousands of studies on virtues, strengths, positive emotions, and positive communities and workplaces. We see this explosion of studies in reviews and meta-analyses on signature strengths and virtues (Schutte and Malouff, 2019), helping behavior (Lefevor et al., 2017), creativity (Acar et al., 2020), resilience (Liu et al., 2020), positive affect and broaden and build theory (Fredrickson, 2013), forgiveness (Wade et al., 2014), flow (Harris et al., 2021), gratitude (Boggiss et al., 2020), self-compassion (Wilson et al., 2019), passion (Pollack et al., 2020), mindfulness (Fjorback et al., 2011), hope (Griggs, 2017), optimism (Rozanski et al., 2019), meaning in life (Manco and Hamby, 2021), volunteering (Milbourn et al., 2018), positive forms of motivation (Ntoumanis et al., 2021), value affirmation (Howell, 2017), school-based positive interventions (Tejada-Gallardo et al., 2020), workplace positive interventions (Donaldson et al., 2019), and other forms of positive intervention (Carr et al., 2020). This research has led to a broadening of the definition of positive psychology itself, with theorists arguing that positive psychology needs to include both positive and negative constructs and needs to consider a wider number of methodologies and levels (e.g., group, culture, etc.; Lomas et al., 2021).

The proliferation of effective positive psychology interventions is exciting, but the very growth and breadth of the field makes it hard to learn and to synthesize in a way that casts a clear light on next steps. Looking back, we see clear progress, but the best pathway forward is far less clear. How do we make positive psychology interventions stronger and more effective for more people?

A common-sense approach might lead the field to follow in the footsteps of clinical psychology and pit a variety of new interventions against old interventions, and see which one “wins”, i.e., is more effective. However, this model has failed to improve interventions in clinical psychology (Johnsen and Friborg, 2015; Ljótsson et al., 2017; Jones et al., 2019) and there are few reasons to believe its impact on positive psychology would be any different. Standing still also seems unwise, because despite decades of effort designed to promote well-being, there is no clear evidence that well-being is increasing in the world (Easterlin and Angelescu, 2009; Richter et al., 2019; Marquez and Long, 2021). A alternative approach is needed.

We argue that the field of positive psychology needs to make two major shifts to continue its progress. The first is a shift from evaluating complex intervention packages to evaluating specific intervention elements that target processes of change (Hayes et al., 2020a). We need to know why positive psychology interventions work in a more granular way that can help us refine and target our interventions. Second, we need to stop assuming that the same intervention will have the same effect on all people. We need to tailor interventions for particular people in particular contexts (Hayes et al., 2019). The present paper will provide a framework for understanding and implementing process-based, personalized positive psychology interventions in a way that builds on the best of the existing knowledge base in our field.

## From Evidence-Based Packages to Processes

In the early stages of a field, it makes sense to test complex intervention packages against waitlist and active controls. We use this “package” approach because, initially, we do not know if the interventions will produce meaningful effect sizes and it seems better to be comprehensive to maximize the chance that interventions will produce meaningful change. After two decades of research, however, we now know positive psychology interventions produce meaningful effect sizes, often in the “medium” range (Donaldson et al., 2019; Schutte and Malouff, 2019; Boggiss et al., 2020; Carr et al., 2020; Liu et al., 2020; Manco and Hamby, 2021; Ntoumanis et al., 2021). The question now is, will continuing to evaluate complex packages make us better at promoting well-being?

The answer is likely “no.” The package approach does not allow us to identify what elements in the package are active, which is not only inefficient, it also slows conceptual progress. A complex package may, overall, produce benefits, whilst also having many unnecessary elements. For example, Fordyce's (1977) happiness program has many distinct elements, such as improving organization, increasing creativity, helping people to value happiness, and developing a healthy personality or character. We have little evidence that all these elements are equally valuable or necessary. This is not a criticism of the Fordyce program—rather, it is an acknowledgment of the limit of our approach to evaluating it. Assessing the effect of entire packages does not let us assess the importance of the components within it, nor the process of change they engage. As a practical result, we may feel pressured to administer the entire package or, if time or resources do not permit, choose elements from the package that we intuitively think might work best. This is not a good way to make either scientific or practical progress.

The package approach makes it difficult to refine and compare interventions, because we don't know what is being compared in a deeper or more functional sense. Perhaps packages with different names are really altering similar processes (Wolitzky-Taylor et al., 2012) or two similarly named programs (say, two “mindfulness” interventions) are targeting unique processes. When we don't know what we are comparing, it is unlikely that comparisons will be useful. When we don't test the conceptual basis of interventions with precision, new approaches will be less able to build on the empirical results of the past.

This appears to be part of why comparing packages in clinical psychology (Arch et al., 2012; Forman et al., 2012) has led to no clear winner (Cuijpers et al., 2020). As a result, some have argued that all interventions work, regardless of the components (Budd and Hughes, 2009), but this leaves the field rudderless, and fails to acknowledge that interventions can work for completely different reasons.

A distinct advantage that positive psychology has over traditional research in clinical psychology is that it has escaped the focus on syndromes and hypothesized latent disease entities. This is a huge step forward in empowering more precise and personalized targeting of changes that matter, but taking advantage of that opportunity requires a research program that is equally well-focused. We can meet this opportunity by shifting from the study of evidence-based packages to evidence-based processes of change and the intervention elements that move them (Hayes and Hofmann, 2018; Hofmann et al., 2021).

We define processes of change as theory-based, dynamic, progressive, contextually bound, modifiable, and multilevel change mechanisms that occur in predictable, empirically established sequences oriented toward desirable outcomes (Hofmann and Hayes, 2019). Let's break this definition into its parts.

### Theory Based

Processes are based on clear, testable scientific theories of positive influence. For example, Self-determination theory provides clear, testable theories that increasing autonomous motivation in the workplace will lead to increased productivity (Manganelli et al., 2018).

### Dynamic

Processes can be bidirectional, interactive, and non-linear. For example, challenges to one's life may become too great to assimilate, and then change may involve sudden disturbance and increased variability that leads to dramatic transformation (Hayes et al., 2007). Trauma can lead to initial distress and destabilizing followed by post-traumatic growth, such as an increased appreciation of life, more meaningful relationships, and increased sense of personal strength and meaning (Tedeschi and Calhoun, 2004).

### Progressive

Therapeutic change processes may need to be ordered in particular ways to produce optimal effects. For example, increasing self-esteem may be a useful precursor to increasing self-compassion (Donald et al., 2017), if a person who feels they are worthy of compassion is more willing to treat themselves kindly.

### Contextually Bound and Modifiable

We need processes that are going to be useful to the practitioner and thus must start with the contextual factors practitioners can actually change. Contextual knowledge is doubly useful because we also need to deploy processes that are sensitive to context features of the client's life (Ciarrochi et al., 2016). For example, skillful practitioners can increase the extent that people think in broader and more useful ways in a difficult situation (Kazantzis et al., 2018). This evidence makes reappraisal a potentially strong focus of change, and potentially more useful than something that is difficult to modify, like personality or temperament. However, this process needs to be considered in context, as reappraisal may not be useful for all people in all situations. For example, youth may be less able to use reappraisal strategies than adults (Brockman et al., 2017). Similarly, the precise nature of the contextualized skill needs to be known. For example, we know that reappraisal is most helpful when it fosters cognitive flexibility (Arch et al., 2012).

### Multilevel

Processes are nested within others. Consider, for example, the construct of hope, or one's sense of agency and efficacy in identifying pathways for achieving one's goals (Snyder et al., 1991). We can examine hope on the physiological, psychological, or social level. At the physiological level, hope may be associated with higher heart rate variability (Schwarz et al., 2003; Oh and Chae, 2018). At the psychological level, self-reported hope is associated with individual well-being and achievement (Ciarrochi et al., 2007, 2015). Finally, groups can be more or less hopeful, and group level hope may increase the hope of individuals within the group (Parker et al., 2015). Thus, a hope intervention (Snyder et al., 2002) may focus on biological, psychological, and social functioning.

## From Generic to Personalized Interventions

The second issue with the proliferation of positive psychology packages is practical. What intervention do we use for a particular individual in a particular context? For example, if we find that a loving-kindness intervention increases well-being in a group of people, should we assume that this intervention will work for each individual client? Metaphorically, do we treat an evidence-based positive psychology package as a pill that injects positivity into each client's life (Farias and Wiholm, 2015).

Because researchers have tested almost all positive psychology interventions using classical group comparison statistics, our current knowledge base carries the assumption that the average effect at the group level applies to the individual development over time of persons in the group, at least probabilistically. It has been accepted in the physical sciences for 90 years (Birkhoff, 1931) that such an assumption is legitimate only if the modeled events are “ergodic” (Molenaar, 2004). For example, we might assume that the effect of loving-kindness on a group of participants applies to each individual in the group over time, at least probabilistically. If the loving kindness intervention raises happiness by 1 point (on a 10-point scale) over the control group, we might assume that most individuals will experience a 1 point increase while some experience a 3-point increase, and others may experience a 2-point decrease. It turns out that between person variation cannot be used to predict or model within person variation unless the phenomena being modeled are ergodic but that assumption is violated in psychological areas (Hayes et al., 2019) because ergodicity requires both that the phenomenon studied is stationary and that the same dynamic model applies to all persons. A violation of ergodicity means far more that the well-worn knowledge averages do not apply to individuals—-rather it means that group effects cannot be used to model individual change even probabilistically.

A solution to this problem is to examine psychological phenomena using experience sampling and similar high temporal density approaches, and to analyze relationships within individuals over time. These idiographic relationships can then be aggregated nomothetically, but only if such nomothetic generalizations increase the precision of idiographic findings, or what we have termed an “idionomic” approach (Hayes and Hofmann, 2021).

Idionomic research and analysis is particularly well-suited to examining processes of change which, by definition, are not stationary and thus cannot be properly modeled using classical statistical approaches without violating their underlying assumptions. When applied to processes of change, idionomic research avoids the ergodic error and allows more personalized modeling of intervention impact. We believe that shifting from a package-based approach to a process-based approach will increase the effectiveness of positive psychology interventions.

At this stage of development we can start with what we have learned from the package approach: What are the processes that are likely to be generally effective in groups of people. Examples might be promoting hope, meaning, positive activities, kindness, self-compassion, and identification of strengths. We know all these processes form the core of effective interventions. As a practitioner, our next step is to create a *personalized intervention*: What processes are most important to a particular client, at this time given this goal in this context. We need to create a case conceptualization based on idiographic and idionomic data (Hayes et al., 2020b). We can then target these biopsychosocial processes of change with specific elements or components.

We earlier mentioned that one of the greatest strengths of the positive psychology field is that it discouraged giving people a diagnosis of a syndrome or disorder, using something like the Diagnostic and Statistical Manual of Mental Disorders (DSM). The traditional DSM approach to diagnosis is neither valid nor particularly useful to practitioners (Hayes et al., 2020b). For example, using DSM, we might conclude that two people have the same “disorder,” if they have 7 of 10 symptoms of generalized anxiety. The problem with this approach is the two clinically anxious people may be anxious in entirely different ways and require radically different treatments. The first person might have experienced a traumatic attack; the second person the loss of a job. Would we assume that they should receive the same intervention? Nobody practicing personalized psychology would make this assumption.

A strength-based approach is less likely to fall into the same error, but it can occur. Imagine two people want to engage in more physical activity. The first-person experiences substantial positive affect, but struggles to stay organized and committed to long-term goals. The second person is extraordinarily organized, but experiences little motivation or positive affect. These two people may need very different interventions. Perhaps the former would respond better to behavioral activation and goal setting, while the latter may need more focus on value clarification and motivation.

This is the strength of a process-based approach. There is no reason to give individuals the same packaged intervention. We don't have to think of someone who struggles to engage in activity as having a deficiency or disorder. Instead, we can identify what drives activity for that individual, leaving behind the demonstrably false assumption that the same processes drive activity in all individuals.

## A Unified Framework

### The Extended-Evolutionary Meta Model

Perhaps the biggest step in creating a unified positive psychology is agreeing on terms. This is a challenge, because there are hundreds of theories and measures in positive psychology, each often using their own terms. Consider the list of terms in positive psychology that link to positive thinking: Hope, optimism, primal beliefs, positive reappraisal, confidence, self-concept, self-esteem, self-efficacy, learned helplessness, reframing, positive problem orientation, automatic negative thoughts, and so on and on. This problem is not specific to positive psychology, it haunts all of psychology. Researchers call it the “jingle-jangle” fallacy: terms often sound different but are the same, or sound the same but are different (Marsh et al., 2003).

One way to proceed would be to work within one broad, theoretical framework and seek to integrate all terms within that framework. However, if we were to choose the terms of one theory, say Seligman's PERMA theory of well-being (Seligman, 2011), then we risk seeming to be irrelevant to self-determination theory (Ryan and Deci, 2017) or broaden-and-build theory (Garland and Fredrickson, 2013). In addition, any set of terms located within a current theory risk being irrelevant as new theories arise, as new terms are needed, or as old theories fall away. For example, should we integrate the new area of “Primal World Beliefs” (Clifton and Yaden, 2021) into one of the older theories, or does it need a new theoretical framework with new terms?

A field cannot progress without shared terms and assumptions (Alexandrova, 2017). We propose to use a meta-theory as an organizing framework, the Extended Evolutionary Meta-Model, or EEMM (Hayes et al., 2020a,b). Evolution is the foundation of all life sciences, and perhaps for this reason, few major psychological theorists argue that their views are hostile to an evolutionary account. Take PERMA, SDT, and broaden and build theories as an example: each of the well-known developers of these views have written about the importance of evolutionary theory to their thinking (Cohn and Fredrickson, 2006; Seligman et al., 2006; Seligman, 2011; Ryan and Deci, 2017).

We can apply modern evolutionary science principles not just to genes, but also to epigenetics, behavioral learning and symbolic thought (Jablonka and Lamb, 2006). Evolution applies to the individual and groups; it applies across disciplines and cultures. It is well-suited to serving as the foundation for promoting positive behavioral and cultural change (Wilson et al., 2017). Importantly, modern multidimensional and multi-level evolutionary principles can be used in a prosocial way that avoids any whiff of eugenics or social Darwinism (Wilson et al., 2017). Evolutionary principles can be readily linked to context and purpose and are used for the purpose of promoting equality, reconciliation, peace, prosocial behavior, and meaningful living (Wilson et al., 2017).

The core driving force behind evolutionary change are the principles of variation, selection, and retention, in a specific context (Wilson et al., 2017; Hayes et al., 2020b). Without variation, behavior change is impossible. Indeed, some positive psychology interventions focus specifically on inducing healthy variation. For example, a positive psychologist might induce people to experience positive affect (Lyubomirsky et al., 2005; Boehm and Lyubomirsky, 2008), so they are more likely to be exploratory, socialize, and build skills and resources (Fredrickson, 2001). Similarly, mindfulness is expected to broaden awareness and increase variability (Garland and Fredrickson, 2013). Psychopathology is often characterized by rigidity over flexibility (Hayes et al., 1996), and approaches like Acceptance and Commitment Therapy deliberately seek to increase values-based behavioral activation and exploration, even in the presence of distress (Hayes and Ciarrochi, 2015; Hayes, 2019).

We can think of *selection* as the goal of the intervention or desired behavior. It is what works for the client in a particular context. The client may want to achieve a goal, develop meaning, connection, joy, curiosity, health, or live in a valued way. Practitioners evaluate every intervention in terms of how it serves the client's needs and values, both short term and long term. For example, we can think of the needs addressed by self-determination theory as selection criteria for processes of change (Hayes, 2019). A self-determination practitioner might ask, does this process support the clients need for autonomy, competence, and/or connection?

Retention relates to how we maintain adaptive behavior. Many positive psychology interventions promote retention through maintenance, follow up, homework, practice, broadening psychological patterns, or habit formation (Kazantzis et al., 2018).

We set variation, selection, and retention inside a particular context, because no process of change is useful without considering the context. Sharing emotions may be adaptive at home, but harmful in some work environments. Context includes the client's current situation, history, environment, repertoire, and goals. Positive psychologists often discuss context in terms of stimulus control, social support, culture, and so on, but also discuss context in terms of the environments and social groups that people select and/or shape. Action and context are intertwined, and a modern evolutionary approach recognizes that people are not passive responders to the environment, but actively select, change, and create the niches in which life unfolds. For example, job crafting interventions encourage people to redesign the workplace to be more need satisfying (Tims et al., 2013). Mate selection assistance or relationship enhancing skills help people create more supportive social niches in which to develop their lives (Siette et al., 2017; Hielscher et al., 2021; Veronese et al., 2021).

We can cast every intervention and every process of change in terms of variation, selection, and retention, in context. For example, imagine you are working in an educational context, with a specific student who is procrastinating and consistently does not feel smart enough to achieve their academic goals (low and rigid self-concept), and does not have a clear sense of why they are attending the university in the first place (low, or unclear motivation) and feels incompetent, with no sense of academic self-efficacy. The student does not complete work on time and is in danger of dropping out and something must change to prevent dropout – that is the outcome being targeted – but the hypothesized processes of change in this case might be rigid and low self-concept, unclear academic motivation, and a lack of a sense of behavioral competence and self-efficacy.

The practitioner might induce healthy variation in low self-concept by encouraging the client to engage in something new, such as a mindfulness intervention designed to help notice and let go of negative self-talk and thus counteract the low self-concept. Let's say we implement the intervention, but after a week or two, the student has made no noticeable improvements in self-concept or procrastination. Since this variation focused intervention did not in fact lead to healthy variation in self-concept, it is not surprising that procrastination did not change. Suppose the practitioner now shifts more toward low or unclear motivation and low sense of competence and self-efficacy.

She now encourages the student to engage in mental contrasting (Clark et al., 2021), which involves asking the student to consider and elaborate on the most positive outcome they associate with achieving their goal, and also to elaborate on important obstacles to the goal. A week later, we discover that mental contrasting has increased academic motivation and self-efficacy and reduced procrastination. The very success of this intervention suggests that, for this person, motivation and self-efficacy are key processes of change and mental contrasting is a key intervention. The practitioner might then work with the student to make mental contrasting a habit (retention) and to monitor issues of motivation and self-efficacy going forward. This simple example illustrates how variation, selection, and retention can be used to personalize an intervention. With another client, or in another context, perhaps mindfulness would have worked, but not mental contrasting.

The same simple principles of variation, selection and retention can apply to more complex problems. For example, imagine that a client just got promoted, and now struggles with work-life balance. They feel constant time pressure to finish everything on time, but they want to do everything perfectly so nobody could ever criticize them. As a result, they are sacrificing quality time with their children and partner. Although this problem is common, it is unlikely to have a common solution. Everyone's context and demands are likely to vary in important ways. The skillful practitioner will thus seek to identify what the problem is and what the client needs. To keep things simple, here we will assume the client is overly perfectionistic at work and has overcommitted to workplace values vs. home values. We might seek to put several processes into play, such as acceptance of distress at doing something imperfectly, clarification of work/life values, increased mindfulness, and presence at home, and improved commitment to sleep and exercise to manage physiological stress. Each of these interventions might introduce something new into the client's life and would be selected and retained if it altered targeted processes of change and resulted in improved work-life balance.

### Organizing Evidence-Based Processes

**Figure 1:**
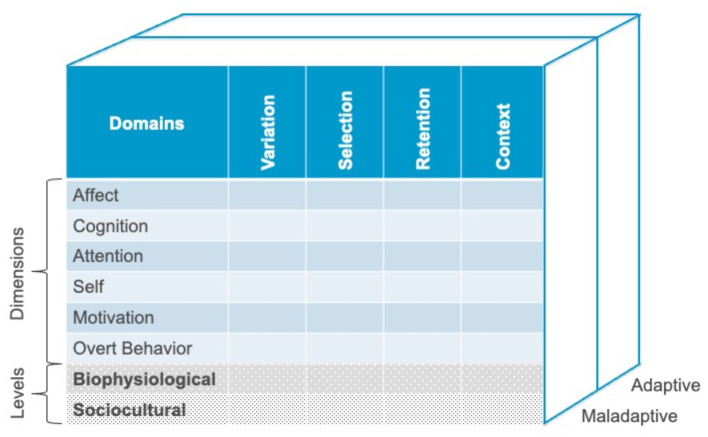
A conceptual space for the examination of adaptive and maladaptive change processes, based on modern multi-dimensional, multi-level evolution science. Copyright Steven C. Hayes, Stefan G. Hofmann, and Joseph Ciarrochi. Used by permission.

The processes of variation, selection, retention, and context can be applied on multiple levels and multiple dimensions, and each of these dimensions can be targeted for variation, selection, and retention, in a particular context. Figure 1 presents an overview of the model.

The extended-evolutionary meta model proposes six psychological dimensions: Affect, cognition, attention, self, motivation, and overt behavior. We identified these dimensions based on a comprehensive review of processes of change in clinical interventions (Hayes et al., 2020a). The processes could readily be sorted into these categories or their combination. We did not assume any hard and fast distinctions – rather we found the dimensions to be useful. Besides the psychological level, processes of change can occur at the sociocultural level of analysis or the biophysiological level. In addition, processes can be empirically shown to be adaptive or maladaptive.

In what follows, we discuss the process of change that researchers have proposed in positive psychology. We identified these processes from literature in several ways: First, we used the positive psychology expertise of Ciarrochi and Oades (first and third author). Second, we identified process *via* a recent bibliographic analysis of positive psychology (Hendriks et al., 2019). Finally, we reviewed the construct topics of every article published between 2016 and 2021 in the Journal of Positive Psychology. We sought to include every major process, in some form, in the positive process column of Table 1. The negative processes come from prior clinical reviews (Ciarrochi et al., 2021). Appendix A contains a table of major positive processes and linking research articles.

**Table 1:** Evidence-based processes classified by dimension.

**Dimension**	**Undermining negative processes**	**Promoting positive processes**
Affect	Reducing negative affect *Mindfulness*: Non-reactivity to negative emotion acceptance of negative feelings	Increasing positive affect, happiness, feelings of curiosity or awe. *Nonattachment:* Letting go of pride and need to hold on to positive feelings, when doing so is beneficial Emotional intelligence: emotion identification and management Promoting contentment, tranquility Resilience and coping (focused on emotion rebound from challenge) Love
Cognition	*Mindfulness:* non reactivity to thoughts; disengage or defuse from unhelpful thoughts. Reappraise or reframe negative thinking. Challenge dysfunctional thinking. Disrupt excessive worry or rumination. *Mental contrasting*: identify barriers to achievement	Seeking accurate information, effective problem solving; Developing positive or helpful self-talk, reappraisal, optimism, attitudes, hope. *Job crafting*: Redesigning job; problem solving Solution focused interventions Positive imagery. Recalling positive memories; imagining positive futures *Mental contrasting*: Identify benefits of goal achievement Well-being literacy Wisdom Creativity (divergent thinking), open mindedness
Attention	*Mindfulness:* Awareness of negative feelings, acting with awareness of negative consequences of behavior Reduce focus on task irrelevant thoughts. Reducing attentional bias to negative information	*Mindfulness*: awareness of positive consequences of behavior. Savoring: Embracing present moment, Focusing on things I'm grateful for. Focusing on the positive. Flow: full attentional absorption in task Noticing nature
Self	Undermining negative self-concepts, low self-esteem, low self-efficacy., Undermining fixed mindset. *Self-compassion*: seeing oneself as suffering and deserving compassion	*Identifying strengths:* focused on skills and capabilities Growth mindset; positive self-esteem and self-efficacy. Promoting a transcendent sense of self. Best possible self-interventions *Self-compassion*: Seeing self through the eyes of a friend Spirituality Post-traumatic growth, authenticity
Motivation	Undermining amotivation, and unhelpful aversive motivation (e.g., escape from guilt, pressure)	*Identifying strengths*: focused on what love and value. Promoting positive forms of motivation, e.g., supporting autonomy, connectedness, and competence needs. Value clarification, and/or promoting recognition of passions and preferences *Job crafting:* Improving fit between own needs and job Promoting people to value humility, patience, courage, gratitude, responsibility, and other aspects of character Personal strivings (identifying motivation for)
Overt behavior	Undermining unhelpful behavior and habits. Promoting perseverance (persistence in behavior in presence of negative affect or pain).	Promoting positive behavior and habits. Promoting patience (persistence in absence of immediate reward). Increasing pleasurable or fun activities. Goal setting and planning skills Courageous behavior *Job crafting:* Altering work environment and behavior Promoting leisure activity and/or physical activity
Social level/dimension not specified	Reducing loneliness, conflict, social skill deficits	Promoting social connection Civic engagement Promoting prosocial behavior Relationship with animals Help seeking Couple resilience

We roughly sorted processes in terms of the EEMM (Table 1). These processes cross-multiple dimensions, so this classification can be viewed as a starting point for discussion. For example, wisdom, post-traumatic growth, spirituality, and mindfulness are likely to cross multiple dimensions (Reker et al., 1987; Wong, 1998; Linley and Joseph, 2004).

We will focus our discussion on the positive processes here, as researchers have reviewed negative processes in the clinical literature (Hayes et al., 2020a). Most of the positive processes in Table 1 have evidence supporting their value (see Appendix A), though all of them need additional evidence, especially for determining whether the process is useful in predicting change at an individual level. Said in another way, the contextual nature of the EEMM emphasizes that all “positive” or “negative” processes of change need to be placed in scare quotes in terms of their impact for a particular individual, which is more based on the idiographic network of relations than overall nomothetic expectations (e.g., cross-sectional correlations).

The reader may notice that terms that often suggest one process, e.g., mindfulness, or self-compassion, often consist of multiple rows in the EEMM. For example, mindfulness can be linked to positive and negative cognition, affect, attention, and sense of self. Thus, different programs that utilize mindfulness interventions may alter processes of change in a variety of dimensions that may produce substantially different outcomes. Strength spotting too consists of multiple components, such as building one's sense of self (*courage is your strength*) and developing motivation (*your love of learning is a strength*) and the same caution applies. The EEMM is more of a reminder of dimensions and domains that can be addressed than it is an ontological periodic table.

Table 1 provides a broad overview of the field, cast in terms of the EEMM and targeted processes of change. Clinical psychology has largely focused on processes in the left-hand column and has emphasized undermining of maladaptive processes that lead to mental health problems. Positive psychology has largely focused on processes in the right column and promotive adaptive processes that lead to flourishing. Naturally, both fields target both kinds of process, so the difference is primarily emphasis. One thing appears certain: We cannot reduce “positive” and “negative” to a single dimension (Diener and Emmons, 1984; Monni et al., 2020). Studying mental health problems will not lead you to understand flourishing, and vice versa (McGaffin et al., 2015). In a given day, people can engage in high amounts of maladaptive and adaptive behavior (Ciarrochi et al., 2021), and experience high levels of both positive and negative affect (Crawford and Henry, 2004). Or they can engage in little behavior with adaptive significance or experience little affect of any sort. Adaptive and maladaptive processes are separable.

“Separable” does not mean unrelated. Instead of thinking of positive and negative processes as part of a single continuum, we can see them as part of a complex dynamic network that changes over time (Hayes et al., 2019). For example, imagine you have a client named person X who is dissatisfied with their job and is far from flourishing. How might a positive psychology coach intervene using the EEMM system?

**Figure 2:**
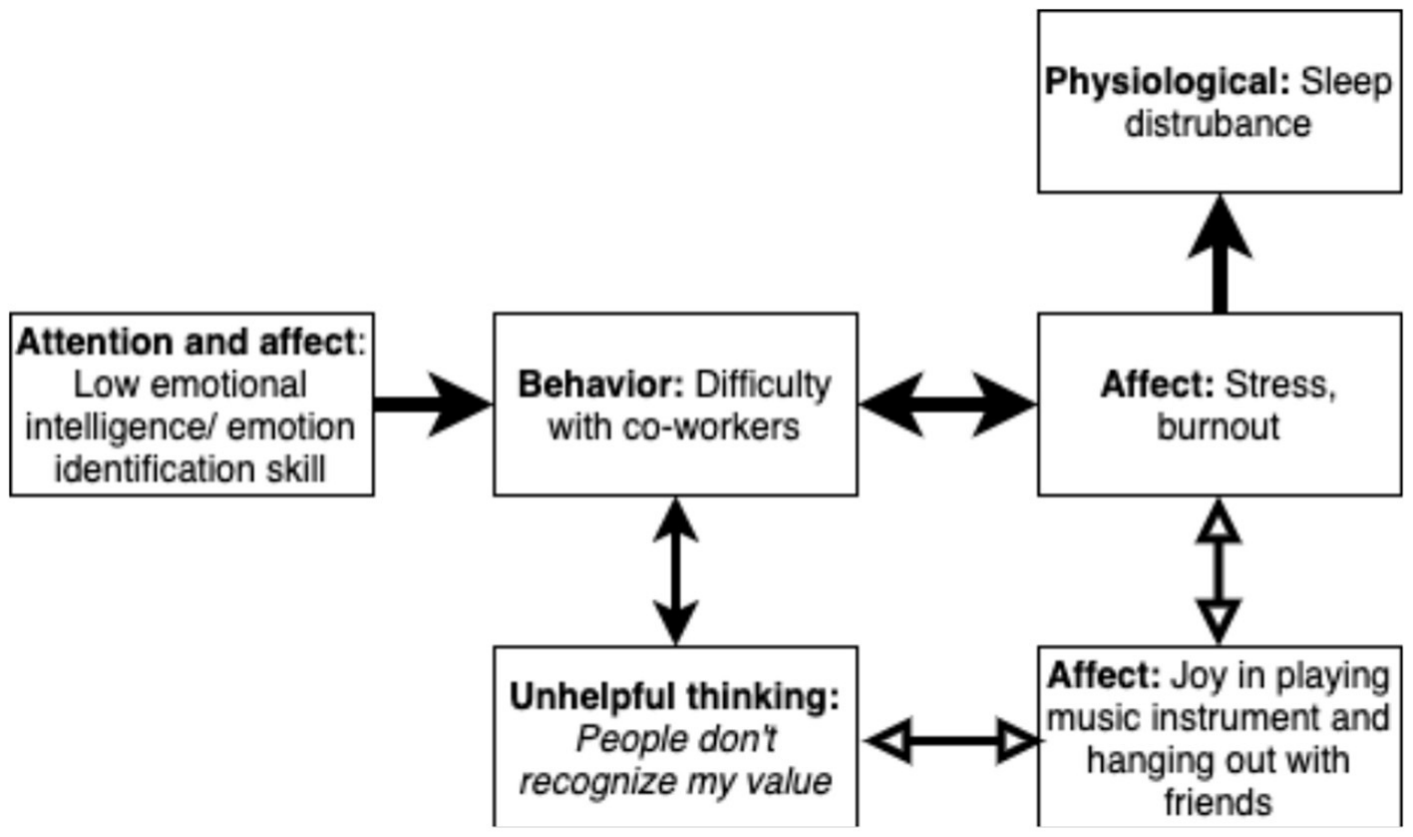
Positive and negative processes as distinct and interacting. Note: Clear arrows indicate negative relationships. Width of line indicates strength of relationship.

We might start with a case conceptualization, as in Figure 2 (Hofmann et al., 2021). Practitioners can draw this out simply by hand on a piece of paper, perhaps after the first session. The conceptualization is not set in stone, but rather speculative and revised as the practitioner works with the client. Black arrows indicate processes that are positively linked (increase in one hypothesized to lead to increase in another). Clear arrows are negative relationships.

Let's say Person X seeks coaching from you for sleep disturbance. You might be tempted to teach them sleep hygiene or help them get to bed earlier, but if the Figure 2 case conceptualization is correct, this is unlikely to work, because sleep problems are a consequence, rather than a cause of problems in their life. Person X has a poor understanding of their emotions and is unaware when they are feeling irritable and anxious. As a result, they are sometimes reactive and impatient with co-workers. This, in turn, causes the co-workers to respond badly to them. Person X responds with unhelpful thinking and burnout. One thing in the network offsets the burnout: Playing guitar with friends. On days when person X does this, they experience less burnout and unhelpful thinking. Note the bi-directional link. This means that on days when person x is under stress, they are less likely to play a musical instrument. This captures one of the great ironies in positive psychology: People may be least likely to do something positive for themselves when they are in most need of it (Ciarrochi et al., 2002; Sheppard et al., 2018).

Using this network as a guide, the practitioner may intervene in multiple ways. They may help the person identify their feelings and recognize when they are feeling irritable and impatient. This may reduce difficulties with co-workers which, in turn, may reduce negative thinking and burnout and increase positive activity. They may work with unhelpful thinking, increase the extent the person engages in pleasurable activities, or teach them more optimal ways to manage difficult co-workers.

So far, this shows a conceptual and idiographic analysis that can help personalize interventions. There can also be data-driven analysis, based on experience sampling measures (e.g., Ciarrochi et al., 2021) and network analyses (Gates et al., 2014; e.g., see Sanford, 2021). There appears to be no conceptual barrier to implementing a process-focused network approach to case conceptualization and treatment (Hayes et al., 2019; Hofmann et al., 2021).

What positive psychology processes might go into this network? Table 2 presents some of the most common positive psychology constructs, categorized by the EEMM. Notice how each construct is linked to processes and set at a particular level or multiple levels (L in the table). Note also that some constructs include multiple dimensions. There may be controversy about what processes are associated with a construct, but the EEMM classification system at least allows us to argue and discuss perceived differences using the same language. This is a first, critical step to improvement.

**Table 2:** Positive psychology constructs and associated processes in positive psychology.

**Positive psychology construct with example interventions**	**L**	**Af**	**C**	**A**	**S**	**M**	**B**
**Optimism and hope**. Think of time when lost something, and a door closed and another opened (Seligman et al., 2006)	I	x	x				
**Strengths**. Identify what you are good at, identify skills, abilities, passions; find ways to apply strengths to everyday life (Schutte and Malouff, 2019)	I				x	x	x
**Meaning in life**. Clarifying values, find meaning in adversity (Steger et al., 2008; Manco and Hamby, 2021)	I					x	
**Positive affect/broaden-and-build**. Inducing positive affect to enhance exploration, socializing, skill development (Howell, 2017)	I&S	x					x
**Goal setting**. Make goals specific and measurable. Engage in mental contrasting (think of benefits; think of barriers) and if-then contingency planning (Clark et al., 2021)	I		x				x
**Growth mindset**. Promote idea that growth is possible, and undermine idea that you are fixed (Mueller and Dweck, 1998; Schleider and Weisz, 2018)	I				x		
**Creativity**. Increase fluency, flexibility, originality, and elaboration (Alves-Oliveira et al., 2021)	I		x	x			
**Humor**. Remember three funny things that happened during the day (Wellenzohn et al., 2018)	I	x	x				
**Patience, conscientiousness, grit, and persistence**. Teach people to persist in behavior, in presence of a motivation, or difficult feelings (Roberts et al., 2017). Combine persistence and passion (Credé, 2018).	I	x					x
**Positive self-talk**. Teach people to use helpful language to encourage and direct themselves (Hutchinson et al., 2008; Blanchfield et al., 2014).	I	x	x				x
**Multi-dimensional, mindfulness**. Teach people experiential acceptance, non-reactivity to thoughts, focus on present moment, acting with awareness, observing (Fjorback et al., 2011)	I	x	x	x			x
**Self-determination theory**: Support the need for autonomy, competence, and connectedness (Ryan and Deci, 2017).	I/S				x	x	
**Self-efficacy, self-esteem, and self-concept**. Help people to believe they are effective, skillful, worthy of love, and not broken (Niveau and New, 2021)	I				x		
**Savoring**. Focus on particular experiences, maximize happiness, and physical sensory, emotional, and social experience (Smith and Hanni, 2019)	I	x		x			
**Use of breath**. Slow breathing to induce calm and focus (Zaccaro et al., 2018).	P	x		x			
**Biofeedback** focus on body responses, engage in strategies to reduce stress (Lehrer et al., 2020).	P	x		x			
**Gratitude**. Identify reasons for gratitude. Saying thank you to others (Boggiss et al., 2020).	I/ S	x	x	x			x
**Kindness**. Perform acts of kindness. Prosocial spending (Curry et al., 2018).	S	x					x
**Empathy**. Increase effective communication broaden perception, bridge gap between self and other (Levett-Jones et al., 2019).	S	x	x		x		
**Prosociallity**. Build cooperation, strong teamwork (Mesurado et al., 2019).	S						x
**Job crafting**. Reimagine workplace, identify values in workplace, modify workplace context) (Tims et al., 2013)	I&S		x			x	x
**Positive parenting**. Improve parenting knowledge, motivation, affect, and behavior. (Sanders, 2012)	I&S	x	x			x	x

*L, Level; P, physiological level; I, individual; S, Social; Dimensions: Af, affect; C, cognition; A, attention; S, self; M, Motivation; B, overt behavior*.

Table 2 represents hypothesized processes. These processes must remain hypothesized until future intervention research confirms that the interventions change the process variables. We also need longitudinal analysis to examine if interventions have unexpected benefits on processes. For example, meaning in life interventions may initially motivate people to engage in new behavior. Over time, this may indirectly increase their self-confidence and optimism. This kind of hypothesis highlights the need for intensive, time series data.

Table 2 presents interventions that focus on one construct. There are comprehensive positive psychology packages that are intended to cover most of the major constructs in positive psychology. The EEMM can clarify similarities and differences between these packages too. We illustrate this in Table 3.

**Table 3:** Hypothesized processes in three multi-component, positive psychology interventions.

**Components**	**L**	**Af**	**C**	**A**	**S**	**M**	**B**
**Seligman-based, PERMA intervention (Gander et al.**, **2016****)**							
*Pleasure:* Remember three things you have experienced today that were related to fun, amusement, joy, pleasure.	I	x					
*Engagement*: Remember three things you have experienced today where your attention was particularly focused, and you were not aware of your surroundings.	I			x			
*Positive relationships*. Remember three things you have experienced today that were positive experiences with other people.	S	x					
*Meaning*: Remember three things you have experienced today that were personally significant and meaningful	I	x				x	
*Accomplishment*: Remember three things you have experienced today where you were successful or where you had the impression that you did something really well.	I	x	x		x	x	
**Fordyce' happiness program (Fordyce**, **1977****; Narmashiri et al.**, **2014****; Mirbolouk and Salari**, **2021****)**							
Spend more time socializing	S						x
Develop an outgoing, social personality	S				x		x
Become more active	I						x
Lower expectations and aspirations, be realistic	I		x				
Develop positive, optimistic thinking	I	x	x				
Get better organized and plan things out	I		x				x
Eliminate negative problems (especially stop worrying), decrease concerns	I	x	x				
Become more present oriented	I			x			
Value happiness, think about enhancing happiness	I	x				x	
Increasing creativity	I		x				
Considering yourself, authenticity, be yourself	I				x	x	
Developing healthy personality/character	I				x		
Being productive at work/meaningful work	I					x	x
**Social and emotional learning programs (Taylor et al.**, **2017****; Lawson et al.**, **2019****)**							
*Self-awareness*-Identifying emotions, having an accurate self-perception, recognizing strengths Self-confidence, self-efficacy, recognizing values	I	x	x	x	x	x	
*Responsible decision-making*: Identifying problems, analyzing situations, solving problems, self-evaluation, self-reflection, ethical responsibility.	I/S		x		x		x
*Relationship skills:* Communication, social engagement, relationship building, teamwork, establishing and maintaining healthy relation-ships	S					x	x
*Social awareness:* Perspective-taking, empathy, appreciating diversity, respect for others.	S		x			x	x
*Self-management:* Regulating emotions and behavior.	I	x					x

*L, Level; which refers to physiological (P), I, individual (I); and/or S, Social level (S); Dimensions: Af, affect; C, cognition; A, attention; S, self; M, Motivation; B, overt behavior*.

Although the three packages in Table 3 are all designed to promote well-being, they are clearly different. The package based on Seligman's well-being theory is much more focused on promoting positive affect than the other two packages. Fordyce's program increases positive affect, but also includes components focused on behavior and character. Both programs focus heavily on individual level processes. In contrast, Social and Emotional Learning programs focus more on the social level and are less focused on producing positive affect and more focused on self-regulation. This emphasis makes sense, as educators often implement SEL programs inside a school context, in order to reduce bullying, help students to stay focused and learn, and promote positive relationships between students and teachers.

It is worth noting that whilst these three programs are all designed to be comprehensive, they have many unique components. We might be tempted to combine what is unique in each program into a “meta” program. However, such a program may become too large, and some components might be irrelevant to some people. We speculate that the best way forward would be to identify what a particular individuals needs, in a specific context, and then provide them with the essential components of the interventions that target those needs. Start with the person, not the protocol. For example, if someone is physically inactive and has unrealistic ideas about physical fitness, then this could become the target of the specific intervention for them. If another person is struggling to understand people's motives at work, then the focus of the intervention might involve perspective taking.

The EEMM suggests that successful implementation of the processes in Tables 2, 3 requires a consideration of variation, selection, and retention, in context. It also requires a consideration of the ordering of processes. For example, let's say we want to help someone improve performance in the workplace (context). Assume this person feels stuck and is, at present, unwilling to do anything new. We might start with strength building, to get them doing and thinking in new ways (variation). Once the person believes they have the strength to improve, we might move on to goal setting, encouraging them to try new things at work (variation) and see what improves performance (selection). Perhaps they might try bringing kindness to the workplace or use slow breathing to manage stress and be more present to colleagues, or improve organizational skills, or practice empathy. We would have them monitor what worked and didn't work (selection) and seek to reinforce and make into a habit (retention) what was improving performance.

### Building and Extending on Past Approaches

All models, including the present, have evolved from earlier forms. The EEMM links to the past in four important ways. First, our approach builds from and extends approaches focused on enhancing treatment effectiveness by tailoring treatment to the unique individual and their particular situation (Norcross and Wampold, 2018). Researchers have labeled this approaches as *treatment adaptation, responsiveness, individualizing, personalizing, and tailoring*, and, like the present approach, seek to attune research on processes of change to the details of a specific individual in a specific context.

Second, the EEMM shares the goals of Measurement-based Care (Scott and Lewis, 2015; Lewis et al., 2019), which involves enhancing usual care through systematic evaluation of client data during the intervention process. Ideally, we need to measure important processes that promote change, and these measures should guide action, for example, by warning a practitioner if an intervention is failing to hit its mark. We add to this approach by suggesting that the processes and outcome measures feed into an interactive network, such that processes don't merely affect the outcome in a unidirectional and linear way, but also affect other processes and are influenced by the outcome.

Third, our process model has consilience with many ideas in implementation science. For example, Presseau et al. (2019) suggest that interventions should have a detailed specification of behaviors targeted for change and an alignment between intervention components and outcomes. They argue that we can specify behavior in terms of the Action, Actor, Context, Target, and Time. Our model adds to this approach by suggesting that we can use actions to promote variation, selection, and/or retention, across six dimensions and multiple levels.

Fourth, our approach creates a context for the examination and comparison of specific process-based interventions such as Barlow's universal protocol for transdiagnostic treatment (Barlow et al., 2017; Leonardo et al., 2021) or specific process-based models such as the psychological flexibility model in Acceptance and Commitment Therapy (Hayes et al., 1999). The EEMM is a “meta-model” of evidence-based processes of change that allows specific models and methods to be examined for their completeness while providing a common language and well-accepted framework in the form of evolutionary science for different theorists to communicate what is essential about their approach. Thus, we can examine ACT (Hayes, 2019) as a process-based intervention and the EEMM can be used to see if specific kinds of concepts or methods are needed to expand a traditional ACT approach. Similarly, we can examine the Unified protocol (Barlow et al., 2017) as a set of interventions and if specific areas of the EEMM are not well-covered, efforts can be made to expand the protocol coherently.

We might use the EEMM to examine processes targeted and methods deployed in a positive psychology or other protocol, but the EEMM does not, by itself, suggest a specific protocol or process. It is not possible to compare, say, the PERMA or broaden and build approach to the EEMM, but it is possible to use the EEMM to further develop PERMA or broaden and build as process-based coaching approaches. It is not possible to test the difference between process-based therapy and, say, ACT, but it is possible to compare “off the shelf ACT” and ACT as a form of process-based therapy, and, if outcomes are indeed improved by process-based dynamic tailoring, to see if the improvement is because of larger or broader movement of psychological flexibility processes or other processes that may be suggested by the EEMM.

## Future Directions

Positive psychology has revolutionized the way we approach interventions. It has shifted the focus from fixing problems to promoting thriving, and led to a proliferation of new, positive interventions. Positive psychologists were right to point out that positive functioning is not merely the absence of negative functioning, and vice versa. Indeed, the two often co-occur in the same minute,– anxiety and joy, success and failure, socially skilled and unskilled behavior. These are not necessarily opposites.

This article proposed several steps the field can take to keep making rapid progress. First, instead of focusing on complex packages, the field needs to shift toward evidence-based processes that are often nested within the package. This focus will help us identify the most powerful ingredients in different interventions and combine ingredients in a way that is most effective. If the goal is to build a treatment package that works for many people, models like ORBIT (Czajkowski et al., 2015) could be used to systematically evaluate treatment components, combine them into a proof of concept package, and then conduct pilot and feasibility testing. However, even with packages designed this way, individual assessment is needed as we cannot assume the package will work the same way for each person. Thus, what is needed are models based on idionomic findings that allow the tailoring of intervention kernels within an overall approach that can maximize gains for particular people. The “packages” of the future will not be one-size-fits-all collections, but coherent, flexible models linked to sets of modifiable elements.

Second, we need to consider how negative and positive processes interact to influence individual outcomes. Whilst positive and negative cannot be reduced to a single continuum, we can think of them as interacting processes in a dynamic network (Hayes et al., 2019). For example, positive emotion may help people to manage and recover from stress (Ong et al., 2006). Treating oneself with kindness and compassion may protect people from the harmful effects of low self-esteem (Marshall et al., 2015).

Third, we need to personalize positive psychology. We have made great progress in understanding the ingredients that are likely to promote positive change, but now we need to ask which of these ingredients is most relevant to a particular person in a particular context. For example, whilst promoting positive thinking seems like a universally “good” process, we must recognize the contexts when it is potentially unhelpful, as when focusing on the positive reinforces experiential avoidance (Foody et al., 2013), encourages self-focus and loneliness (Mauss et al., 2012), promotes harmful attachments (Sahdra et al., 2016), or incorrectly focuses blame for a lack of positivity on the person rather than a toxic situation (Ciarrochi et al., 2016).

The practitioner can use the Extended-Evolutionary Meta Model, described here, to target the potential active processes of change identified in both clinical and positive psychology research. A personalized approach would start with case conceptualization, which involves identifying the core processes that are likely to apply to a particular client (Hayes et al., 2020a). Perhaps one client needs more focus on building hope, whereas another needs to develop social skills. The practitioner could then implement a process-based intervention, based on that tentative conceptualization. During the intervention, the practitioner would receive feedback from the client about what is working and not working and would adapt the case conceptualization and intervention accordingly. In this way, the practitioner would implement different, evidence-based processes (variation), select those that are benefiting the client, and then, through practice and habit formation, help the client to retain the skills. The full details of this approach are beyond this paper, but the interested reader can follow up with Hofmann et al. (2021).

Finally, we need to evaluate the utility of the EEMM. We would argue that the model can be shown to be useful if it: (1) addresses the current literature in a way that fits with its assumptions and purposes, (2) builds on existing and well-supported concepts that have precision, scope and depth without arbitrarily leaving aside issues that should be addressed, (3) addresses what is known without distortions or narrow adjustments to deal with anomalies, and (4) leads to new and testable insights. This very paper is an exercise in supporting the first three points. It appears that the EEMM was able to be used to sort the evidence-based processes in positive psychology, without excluding any major theoretical position or approach. In addition, it showed how the positive psychology processes links to clinical psychology processes, demonstrating substantial scope and depth, while challenging the unwarranted notion that clinical psychology and positive psychology are incompatible or cannot be merged. Future research is needed to explore many new research questions, including the possibility that idiographic and process-based tailored interventions may improve outcomes over and above well-established and relatively comprehensive intervention protocols, such as Barlow's Unified Protocol (Barlow et al., 2017). Early research on that question has already yielded suggestive evidence in support of a process-based approach (Fisher et al., 2019).

## Conclusions

There is clear evidence that positive psychology interventions are effective. More people need these interventions. Substantial numbers of people who are failing to flourish and may be classified as “languishing” (Keyes, 2006; Keyes and Westerhof, 2012; Nelson and Padilla-Walker, 2013). About 20–25 percent of people struggle with mental health problems (Baumeister and Härter, 2007), 25 percent of youth experience a traumatic event (Perkonigg et al., 2000), and 50 percent of people experience some form of workplace bullying (Chatziioannidis et al., 2018; Ng and Chan, 2021). Many people who are languishing fail to receive help (Ciarrochi et al., 2002; Cusack et al., 2006; Sheppard et al., 2018).

We believe a process-based approach to positive psychology can help. It may not only lead to more effective interventions, but it may also lead to more people being able to provide help. People don't have to complete postgraduate study in clinical psychology or psychiatry to deploy evidence-based kernels that fit human needs. They don't have to complete specialized training by a guru or have elaborate professional guilds to be able to use evidence-based process. Nobody owns these evidence-based processes, the way people may own packages and training programs targeting hypothetical diseases. We believe many of the evidence-based processes in Tables 2, 3 could be taught to and utilized by everyday helpers: Bartenders, business leaders, hairdressers, coaches, personal trainers, clergy, educators, criminal justice personnel, managers, and youth mentors. With the right technical aids, people engaged in human empowerment could track processes of change and tailor intervention kernels to the process needs of people.

The key would be to focus training on specific intervention kernels linked to specific processes that could be adequately taught to a specific helper in a specific context. For example, youth mentors can learn the most relevant processes for supporting young people to stay engaged in school when emotions arise that might lead to avoidance or withdrawal. They do not need to learn to do more advanced clinical techniques such as exposure therapy. They only need to recognize what they can do, and what is beyond their expertise and needs to be referred to the proper support services.

There will be challenges to training up a broad group of psychological helpers who take a process-based focus. There have been negative effects observed in therapy and mindfulness-based training (Cuijpers et al., 2018; Britton, 2019; Curran et al., 2019). Helpers will need to be taught a core set of skills that will reduce the risk of client alliance ruptures and minimize helper behavior such as rigidity, over control, and delving into areas like past abuse and trauma without adequate knowledge. All of these behaviors may make the client feel disempowered and devalued (Curran et al., 2019). Helpers will probably need technological aides to do the high temporal density assessment required to understand the process-based needs of individuals. Even with these training challenges, however, ending the era of top-down, one-size-fits all protocols and taking a process-based approach will help positive psychology step forward to assume its rightful role in human development world-wide. The problems we suffer as a human community are too great for a handful of specialists to solve alone. The time has come for a more democratic and broadly focused positive psychology that better meets the needs of those we serve.

## Author Contributions

JC, SCH, LO, and SGH contributed substantially to the writing of the manuscript. All authors contributed to the article and approved the submitted version.

## Funding

This research was partially funded by the Australian Research Council (No. LP160100332).

## Conflict of Interest

The authors declare that the research was conducted in the absence of any commercial or financial relationships that could be construed as a potential conflict of interest.

## Publisher's Note

All claims expressed in this article are solely those of the authors and do not necessarily represent those of their affiliated organizations, or those of the publisher, the editors and the reviewers. Any product that may be evaluated in this article, or claim that may be made by its manufacturer, is not guaranteed or endorsed by the publisher.
