# Supplementary material for: Toward a Unified Framework for Positive Psychology Interventions: Evidence-Based Processes of Change in Coaching, Prevention, and Training
Source: Front Psychol. 2022 Feb 10;12:809362. doi: 10.3389/fpsyg.2021.809362 (PMC8866971; doi:10.3389/fpsyg.2021.809362)
Supplement: Supplementary file 1 [file Table_1.docx]

# Appendix A: Research linked to hypothesized positive-psychology processes

|  | **Hypothesized process** | **Current evidence** |
| --- | --- | --- |
| **Affect** | Inducing positive affect increases broaden and build behavior and the development of skill and social networks | (Howell 2017; Fredrickson 2001) |
|  | Non-attachment to positive feelings predicts the development of mental health, and is linked to observed prosocial behavior | (Ciarrochi et al. 2020; Sahdra et al. 2015) |
|  | Happiness and well-being are linked to work performance, positive social relationships, health, engagement in valued activity, and the ability to recover from negative events (resilience) | (Kansky and Diener 2017; Williams, Ciarrochi, and Heaven 2015) |
| **Cognition** | Its beneficial to see problems as a challenge rather than a threat (problem orientation) | (Ciarrochi, Leeson, and Heaven 2009) |
|  | Helping clients to focus on solutions, rather than problems | (Franklin et al. 2017) |
|  | Benefits of teaching social problem solving | (Nezu and Nezu 2021) |
|  | Promoting hope and optimism is associated with well-being, health, and effective behavior | (Rozanski et al. 2019; Ciarrochi et al. 2015; Gallagher, Long, and Phillips 2020; Jiang et al. 2019) |
| **Attention** | Savouring of positive experience is associated with higher well-being and relationship satisfaction | (Colombo et al. 2021; Lenger and Gordon 2019) |
|  | Focusing on gratitude-related experiences is associated with improvements in negative affect and increased happiness | (Dickens 2017; Boggiss et al. 2020; Cregg and Cheavens 2021) |
|  | Mindfulness is linked to a wide range of positive outcomes, including well-being, positive motivation, emotional clarity, and performance. Mindfulness cuts across many dimensions, including affect, cognition, and behavior. | (Donald et al. 2020; Zarate, Maggin, and Passmore 2019; Cooper, Yap, and Batalha 2018; Fjorback et al. 2011; Noetel et al. 2019) |
| **Self** | Best possible-self intervention induces positive affect and optimism | (Heekerens and Eid 2021) |
|  | Self-efficacy is associated with well-being and adaptive behavior | (Jiang et al. 2019; Jiao et al. 2021) |
|  | Having a sense that one can grow and change (growth mindset) is associated with greater achievement, more active coping, and lower distress | (Burnette et al. 2020; Sarrasin et al. 2018) |
|  | Contacting “self-as-context”, or a transcendent sense of self is associated with higher well-being and functioning | (Zettle, Gird, and Webster 2018; L. Yu, Norton, and McCracken 2017) |
|  | Identifying strengths and virtues increases happiness and life satisfaction | (Schutte and Malouff 2019; Gander et al. 2013) |
|  | Self-compassion is associated with higher well-being, better self-regulation of health behavior, and protects against the negative effects of low self-esteem | (Wilson et al. 2019; Ferrari et al. 2018; Biber and Ellis 2019; Marshall et al. 2015) |
| **Overt behavior** | Increasing pleasurable activities increases well-being and reduces distress | (Kanter et al. 2010; Dimidjian et al. 2006; Mazzucchelli, Kane, and Rees 2010) |
|  | Persistence, self-control, and aspects of conscientiousness (competence achievement striving, self-discipline) are linked to well-being and achievement; grit may fall into this group of processes, but has elements of passion as well as persistence | (Credé 2018; Duckworth et al. 2007; Anglim et al. 2020) |
|  | Goal setting interventions promote well-being and goal achievement. Includes cognitive and other dimensions, but specifically focused on behavior. Implementation intentions; mental contrasting (Identify benefit of achieving goal, and potential barriers) | (G. Wang, Wang, and Gai 2021; Epton, Currie, and Armitage 2017) |
| **Motivation** | Harmonious or authentic passion for life activities is linked to lower burnout, higher, well-being and higher performance | (Pollack et al. 2020; Saville et al. 2018; Briki 2017; Vallerand et al. 2007) |
|  | Activities that satisfy the need for autonomy, competence, and connection are associated with higher well-being, performance, and goal attainment | (Nezu and Nezu 2021; S. Yu, Levesque-Bristol, and Maeda 2018; Y. Wang, Tian, and Scott Huebner 2019) |
|  | Values affirmation is associated with well-being, self-control, reduced defensiveness, lower physiological stress, and reduced gap in achievement between mainstream and stigmatized groups. | (Howell 2017; van Koningsbruggen, Miles, and Harris 2018; Jordt et al. 2017; Borman, Grigg, and Hanselman 2016; Armitage et al. 2008; Creswell et al. 2005) |
|  | Motivational interviewing (MI) increases behavior change, adherence to goals, and positive affect. MI includes aspects of overt behavior (e.g., goal setting, etc) | (Huffman et al. 2019; Frost et al. 2018) |

Note: Most processes, as described above, cross multiple dimensions.

**References**

Anglim, Jeromy, Sharon Horwood, Luke D. Smillie, Rosario J. Marrero, and Joshua K. Wood. 2020. “Predicting Psychological and Subjective Well-Being from Personality: A Meta-Analysis.” *Psychological Bulletin* 146 (4): 279–323.

Armitage, Christopher J., Peter R. Harris, Gareth Hepton, and Lucy Napper. 2008. “Self-Affirmation Increases Acceptance of Health-Risk Information among UK Adult Smokers with Low Socioeconomic Status.” *Psychology of Addictive Behaviors: Journal of the Society of Psychologists in Addictive Behaviors* 22 (1): 88–95.

Biber, David D., and Rebecca Ellis. 2019. “The Effect of Self-Compassion on the Self-Regulation of Health Behaviors: A Systematic Review.” *Journal of Health Psychology* 24 (14): 2060–71.

Boggiss, Anna L., Nathan S. Consedine, Jennifer M. Brenton-Peters, Paul L. Hofman, and Anna S. Serlachius. 2020. “A Systematic Review of Gratitude Interventions: Effects on Physical Health and Health Behaviors.” *Journal of Psychosomatic Research* 135 (August): 110165.

Borman, Geoffrey D., Jeffrey Grigg, and Paul Hanselman. 2016. “An Effort to Close Achievement Gaps at Scale Through Self-Affirmation.” *Educational Evaluation and Policy Analysis* 38 (1): 21–42.

Briki, Walid. 2017. “Passion, Trait Self-Control, and Wellbeing: Comparing Two Mediation Models Predicting Wellbeing.” *Frontiers in Psychology* 8 (May): 841.

Burnette, Jeni L., Laura E. Knouse, Dylan T. Vavra, Ernest O’Boyle, and Milan A. Brooks. 2020. “Growth Mindsets and Psychological Distress: A Meta-Analysis.” *Clinical Psychology Review* 77 (April): 101816.

Ciarrochi, Joseph, Peter Leeson, and Patrick C. L. Heaven. 2009. “A Longitudinal Study into the Interplay between Problem Orientation and Adolescent Well-Being.” *Journal of Counseling Psychology* 56 (3): 441–49.

Ciarrochi, Joseph, Philip Parker, Todd B. Kashdan, Patrick C. L. Heaven, and Emma Barkus. 2015. “Hope and Emotional Well-Being: A Six-Year Study to Distinguish Antecedents, Correlates, and Consequences.” *The Journal of Positive Psychology* 10 (6): 520–32.

Ciarrochi, Joseph, Baljinder K. Sahdra, Keong Yap, and Theresa Dicke. 2020. “The Role of Nonattachment in the Development of Adolescent Mental Health: A Three-Year Longitudinal Study.” *Mindfulness* 11 (9): 2131–39.

Colombo, Desirée, Jean-Baptiste Pavani, Javier Fernandez-Alvarez, Azucena Garcia-Palacios, and Cristina Botella. 2021. “Savoring the Present: The Reciprocal Influence between Positive Emotions and Positive Emotion Regulation in Everyday Life.” *PloS One* 16 (5): e0251561.

Cooper, David, Keong Yap, and Luisa Batalha. 2018. “Mindfulness-Based Interventions and Their Effects on Emotional Clarity: A Systematic Review and Meta-Analysis.” *Journal of Affective Disorders* 235 (August): 265–76.

Credé, Marcus. 2018. “What Shall We Do About Grit? A Critical Review of What We Know and What We Don’t Know.” *Educational Researcher*  47 (9): 606–11.

Cregg, David R., and Jennifer S. Cheavens. 2021. “Gratitude Interventions: Effective Self-Help? A Meta-Analysis of the Impact on Symptoms of Depression and Anxiety.” *Journal of Happiness Studies* 22 (1): 413–45.

Creswell, J. David, William T. Welch, Shelley E. Taylor, David K. Sherman, Tara L. Gruenewald, and Traci Mann. 2005. “Affirmation of Personal Values Buffers Neuroendocrine and Psychological Stress Responses.” *Psychological Science* 16 (11): 846–51.

Dickens, Leah R. 2017. “Using Gratitude to Promote Positive Change: A Series of Meta-Analyses Investigating the Effectiveness of Gratitude Interventions.” *Basic and Applied Social Psychology* 39 (4): 193–208.

Dimidjian, Sona, Steven D. Hollon, Keith S. Dobson, Karen B. Schmaling, Robert J. Kohlenberg, Michael E. Addis, Robert Gallop, et al. 2006. “Randomized Trial of Behavioral Activation , Cognitive Therapy , and Antidepressant Medication in the Acute Treatment of Adults With Major Depression.” *Journal of Consulting and Clinical Psychology* 74 (4): 658–70.

Donald, James N., Emma L. Bradshaw, Richard M. Ryan, Geetanjali Basarkod, Joseph Ciarrochi, Jasper J. Duineveld, Jiesi Guo, and Baljinder K. Sahdra. 2020. “Mindfulness and Its Association With Varied Types of Motivation: A Systematic Review and Meta-Analysis Using Self-Determination Theory.” *Personality & Social Psychology Bulletin* 46 (7): 1121–38.

Duckworth, Angela L., Christopher Peterson, Michael D. Matthews, and Dennis R. Kelly. 2007. “Grit: Perseverance and Passion for Long-Term Goals.” *Journal of Personality and Social Psychology* 92 (6): 1087–1101.

Epton, Tracy, Sinead Currie, and Christopher J. Armitage. 2017. “Unique Effects of Setting Goals on Behavior Change: Systematic Review and Meta-Analysis.” *Journal of Consulting and Clinical Psychology* 85 (12): 1182–98.

Ferrari, Madeleine, Keong Yap, Nicole Scott, Danielle A. Einstein, and Joseph Ciarrochi. 2018. “Self-Compassion Moderates the Perfectionism and Depression Link in Both Adolescence and Adulthood.” *PloS One* 13 (2): e0192022.

Fjorback, L. O., M. Arendt, E. Ornbøl, P. Fink, and H. Walach. 2011. “Mindfulness-Based Stress Reduction and Mindfulness-Based Cognitive Therapy: A Systematic Review of Randomized Controlled Trials.” *Acta Psychiatrica Scandinavica* 124 (2): 102–19.

Franklin, Cynthia, Anao Zhang, Adam Froerer, and Shannon Johnson. 2017. “Solution Focused Brief Therapy: A Systematic Review and Meta-Summary of Process Research.” *Journal of Marital and Family Therapy* 43 (1): 16–30.

Fredrickson, Barbara L. 2001. “The Role of Positive Emotions in Positive Psychology: The Broaden-and-Build Theory of Positive Emotions.” *The American Psychologist* 56 (3): 218–26.

Frost, Helen, Pauline Campbell, Margaret Maxwell, Ronan E. O’Carroll, Stephan U. Dombrowski, Brian Williams, Helen Cheyne, Emma Coles, and Alex Pollock. 2018. “Effectiveness of Motivational Interviewing on Adult Behaviour Change in Health and Social Care Settings: A Systematic Review of Reviews.” *PloS One* 13 (10): e0204890.

Gallagher, Matthew W., Laura J. Long, and Colleen A. Phillips. 2020. “Hope, Optimism, Self-Efficacy, and Posttraumatic Stress Disorder: A Meta-Analytic Review of the Protective Effects of Positive Expectancies.” *Journal of Clinical Psychology* 76 (3): 329–55.

Gander, F., R. T. Proyer, W. Ruch, and T. Wyss. 2013. “Strength-Based Positive Interventions: Further Evidence for Their Potential in Enhancing Well-Being and Alleviating Depression.” *Journal of Happiness Studies*. https://link.springer.com/content/pdf/10.1007/s10902-012-9380-0.pdf.

Heekerens, Johannes Bodo, and Michael Eid. 2021. “Inducing Positive Affect and Positive Future Expectations Using the Best-Possible-Self Intervention: A Systematic Review and Meta-Analysis.” *The Journal of Positive Psychology* 16 (3): 322–47.

Howell, Andrew J. 2017. “Self-Affirmation Theory and the Science of Well-Being.” *Journal of Happiness Studies* 18 (1): 293–311.

Huffman, Jeff C., Emily H. Feig, Rachel A. Millstein, Melanie Freedman, Brian C. Healy, Wei-Jean Chung, Hermioni L. Amonoo, et al. 2019. “Usefulness of a Positive Psychology-Motivational Interviewing Intervention to Promote Positive Affect and Physical Activity After an Acute Coronary Syndrome.” *The American Journal of Cardiology* 123 (12): 1906–14.

Jiang, Xinjun, Jingpin Wang, Yanhui Lu, Hua Jiang, and Mingzi Li. 2019. “Self-Efficacy-Focused Education in Persons with Diabetes: A Systematic Review and Meta-Analysis.” *Psychology Research and Behavior Management* 12 (January): 67–79.

Jiao, Xinian, Xiyan Yu, Shuyuan Wang, Zishan Wang, and Zhun Gong. 2021. “Are Effect Sizes in Self-Efficacy Field Changing over Time? A Meta-Meta Analysis.” *International Journal of Psychology: Journal International de Psychologie* 56 (5): 801–11.

Jordt, Hannah, Sarah L. Eddy, Riley Brazil, Ignatius Lau, Chelsea Mann, Sara E. Brownell, Katherine King, and Scott Freeman. 2017. “Values Affirmation Intervention Reduces Achievement Gap between Underrepresented Minority and White Students in Introductory Biology Classes.” *CBE Life Sciences Education* 16 (3). https://doi.org/10.1187/cbe.16-12-0351.

Kansky, Jessica, and Ed Diener. 2017. “Benefits of Well-Being: Health, Social Relationships, Work, and Resilience.” *Journal of Positive School Psychology*  1 (2): | 129-169 |.

Kanter, Jonathan W., Rachel C. Manos, William M. Bowe, David E. Baruch, Andrew M. Busch, and Laura C. Rusch. 2010. “What Is Behavioral Activation? A Review of the Empirical Literature.” *Clinical Psychology Review* 30 (6): 608–20.

Koningsbruggen, Guido M. van, Eleanor Miles, and Peter R. Harris. 2018. “Self-Affirmation and Self-Control: Counteracting Defensive Processing of Health Information and Facilitating Health-Behavior Change.” https://psycnet.apa.org/record/2017-56398-039.

Lenger, Katherine A., and Cameron L. Gordon. 2019. “To Have and to Savor: Examining the Associations between Savoring and Relationship Satisfaction.” *Couple and Family Psychology: Research and Practice* 8 (1): 1.

Marshall, Sarah L., Phillip D. Parker, Joseph Ciarrochi, Baljinder Sahdra, Chris J. Jackson, and Patrick C. L. Heaven. 2015. “Self-Compassion Protects against the Negative Effects of Low Self-Esteem: A Longitudinal Study in a Large Adolescent Sample.” *Personality and Individual Differences* 74: 116–21.

Mazzucchelli, Trevor G., Robert T. Kane, and Clare S. Rees. 2010. “Behavioral Activation Interventions for Well-Being: A Meta-Analysis.” *The Journal of Positive Psychology* 5 (2): 105–21.

Nezu, Arthur M., and Christine Maguth Nezu. 2021. “Emotion-Centered Problem-Solving Therapy.” In *Handbook of Cognitive Behavioral Therapy: Overview and Approaches, Vol*, edited by Amy Wenzel, 1:465–91. Washington, DC, US: American Psychological Association, xxvii.

Noetel, Michael, Joseph Ciarrochi, Brooke Van Zanden, and Chris Lonsdale. 2019. “Mindfulness and Acceptance Approaches to Sporting Performance Enhancement: A Systematic Review.” *International Review of Sport and Exercise Psychology* 12 (1): 139–75.

Pollack, Jeffrey M., Violet T. Ho, Ernest H. O’Boyle, and Bradley L. Kirkman. 2020. “Passion at Work: A Meta‐analysis of Individual Work Outcomes.” *Journal of Organizational Behavior* 41 (4): 311–31.

Rozanski, Alan, Chirag Bavishi, Laura D. Kubzansky, and Randy Cohen. 2019. “Association of Optimism With Cardiovascular Events and All-Cause Mortality: A Systematic Review and Meta-Analysis.” *JAMA Network Open* 2 (9): e1912200.

Sahdra, Baljinder, Joseph Ciarrochi, Philip D. Parker, Sarah Marshall, and Patrick Heaven. 2015. “Empathy and Nonattachment Independently Predict Peer Nominations of Prosocial Behavior of Adolescents.” *Frontiers in Psychology* 6 (MAR): 263.

Sarrasin, Jérémie Blanchette, Lucian Nenciovici, Lorie-Marlène Brault Foisy, Geneviève Allaire-Duquette, Martin Riopel, and Steve Masson. 2018. “Effects of Teaching the Concept of Neuroplasticity to Induce a Growth Mindset on Motivation, Achievement, and Brain Activity: A Meta-Analysis.” *Trends in Neuroscience and Education* 12 (July): 22–31.

Saville, Bryan K., Alex Bureau, Claire Eckenrode, and Michelle Maley. 2018. “Passion and Burnout in College Students.” *College Student Journal* 52 (1): 105–17.

Schutte, Nicola S., and John M. Malouff. 2019. “The Impact of Signature Character Strengths Interventions: A Meta-Analysis.” *Journal of Happiness Studies* 20 (4): 1179–96.

Vallerand, Robert J., Sarah-Jeanne Salvy, Geneviève A. Mageau, Andrew J. Elliot, Pascale L. Denis, Frédéric M. E. Grouzet, and Céline Blanchard. 2007. “On the Role of Passion in Performance.” *Journal of Personality* 75 (3): 505–33.

Wang, Guoxia, Yi Wang, and Xiaosong Gai. 2021. “A Meta-Analysis of the Effects of Mental Contrasting With Implementation Intentions on Goal Attainment.” *Frontiers in Psychology* 12 (May): 565202.

Wang, Yanhui, Lili Tian, and E. Scott Huebner. 2019. “Basic Psychological Needs Satisfaction at School, Behavioral School Engagement, and Academic Achievement: Longitudinal Reciprocal Relations among Elementary School Students.” *Contemporary Educational Psychology* 56 (January): 130–39.

Williams, Kathryn E., Joseph Ciarrochi, and Patrick C. L. Heaven. 2015. “Relationships between Valued Action and Well-Being across the Transition from High School to Early Adulthood.” *The Journal of Positive Psychology* 10 (2): 127–40.

Wilson, Alexander C., Kate Mackintosh, Kevin Power, and Stella W. Y. Chan. 2019. “Effectiveness of Self-Compassion Related Therapies: A Systematic Review and Meta-Analysis.” *Mindfulness* 10 (6): 979–95.

Yu, Lin, Sam Norton, and Lance M. McCracken. 2017. “Change in ‘Self-as-Context’ (‘Perspective-Taking’) Occurs in Acceptance and Commitment Therapy for People With Chronic Pain and Is Associated With Improved Functioning.” *The Journal of Pain: Official Journal of the American Pain Society* 18 (6): 664–72.

Yu, Shi, Chantal Levesque-Bristol, and Yukiko Maeda. 2018. “General Need for Autonomy and Subjective Well-Being: A Meta-Analysis of Studies in the US and East Asia.” *Journal of Happiness Studies* 19 (6): 1863–82.

Zarate, Kary, Daniel M. Maggin, and Amanda Passmore. 2019. “Meta‐analysis of Mindfulness Training on Teacher Well‐being.” *Psychology in the Schools* 56 (10): 1700–1715.

Zettle, R. D., S. R. Gird, and B. K. Webster. 2018. The Self-as-Context Scale: Development and Preliminary Psychometric Properties. *Journal of Contextual*. *10*,64-74.
